# Supplementary material for: New Score for Predicting Results after Catheter Ablation for Atrial Fibrillation: VAT-DHF
Source: J Clin Med. 2023 Dec 22;13(1):61. doi: 10.3390/jcm13010061 (PMC10779542; doi:10.3390/jcm13010061)
Supplement: Supplementary file 1 [file jcm-13-00061-s001.zip › jcm-2728854-supplementary.pdf]

Supplementary data:

Kaplan Meier survival-without-recurrence curves (Figures S1–S5) for each of the five variables

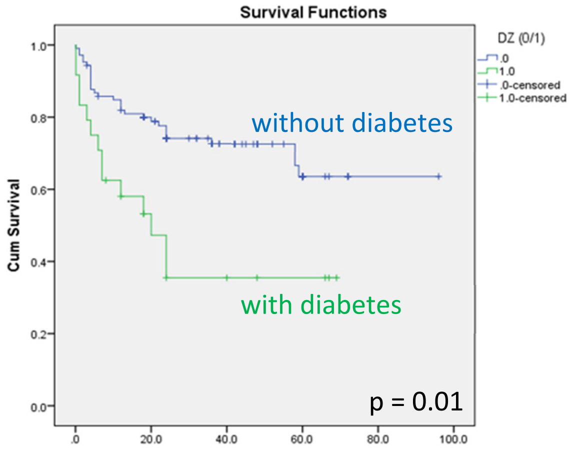

Figure S1. Kaplan Meier for DM (green) vs without it(blue (green).

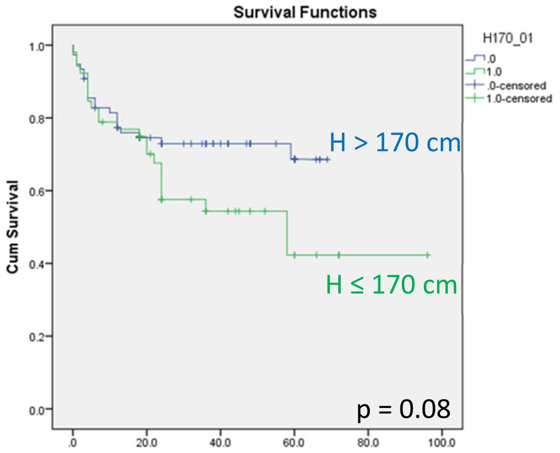

Figure S2. Kaplan Meier for height > 170cm (blue) vs < 170cm

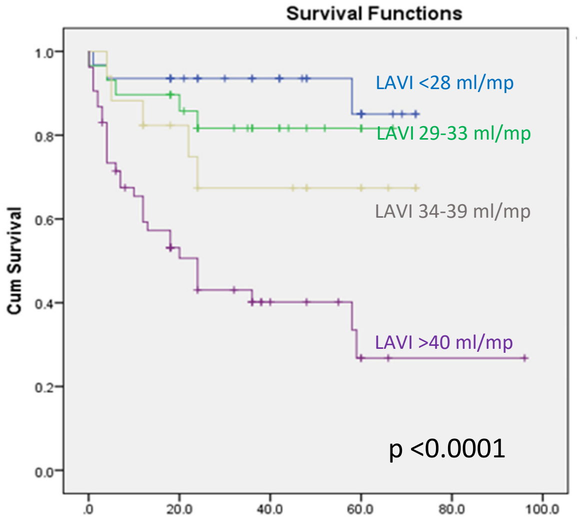

Figure S3. Kaplan Meier for different categories of LAVI (Vol-AS = left atrial volume indexed)

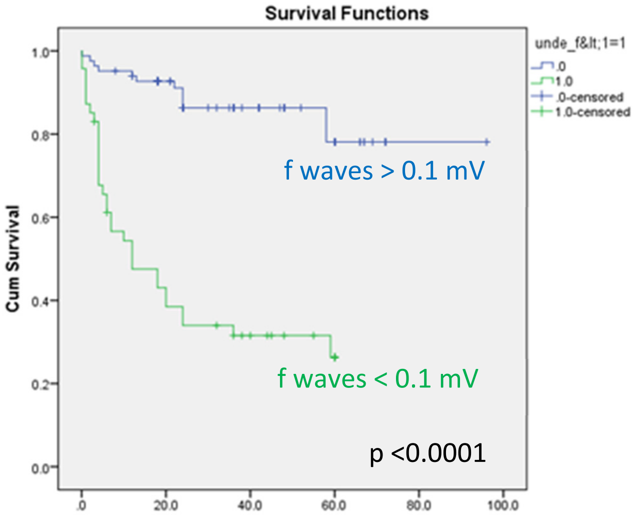

Figure S4. Kaplan Meier for f waves small (green) vs large (blue).

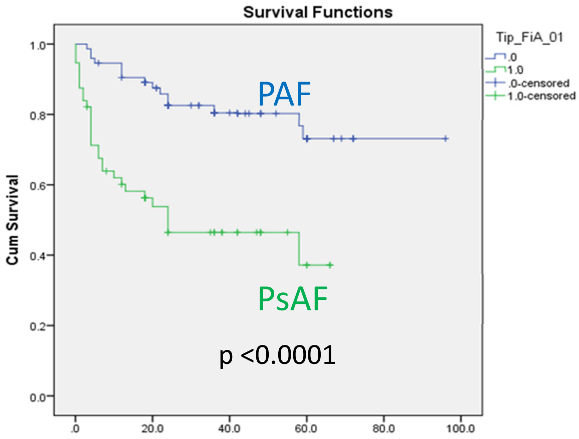

Figure S5: Kaplan Meier paroxysmal AF (blue) vs persistent AF (green)

The cutoff value is 5.875 as shown in the right part of the table below (VAT-DIF is the abbreviation in romanian for VAT-DHF

Coordinates of the Curve

Test Result Variable(s): CHADS-VASC2

Positive if Less Than or Equal To[a]	Sensitivity	1 - Specificity
-1.000	.000	.000
.500	.327	.181
1.500	.639	.398
2.500	.864	.627
3.500	.946	.831
4.500	.966	.940
5.500	.993	.940
6.500	1.000	.988
8.000	1.000	1.000

Coordinates of the Curve

Test Result Variable(s): APPLE

Positive if Less Than or Equal To[a]	Sensitivity	1 - Specificity
-1.00	.000	.000
.50	.500	.143
1.50	.750	.381
2.50	.932	.571
3.50	1.000	.810
4.50	1.000	.952
6.00	1.000	1.000

Coordinates of the Curve

Test Result Variable(s): VAT-DIF

Positive if Less Than or Equal To[a]	Sensitivity	1 - Specificity
-1.0000	.000	.000
.5000	.129	.000
1.1250	.271	.045
1.6250	.341	.045
2.1250	.376	.045
2.3750	.424	.045
2.7500	.435	.045
3.1250	.447	.045
3.3750	.529	.045
3.6250	.576	.091
3.8750	.635	.136
4.1250	.647	.136
4.3750	.682	.159
4.6250	.718	.159
5.0000	.753	.159
5.3750	.776	.205
5.6250	.800	.205
5.8750	.871	.250
6.1250	.871	.273
6.3750	.882	.295
6.6250	.906	.318
7.0000	.929	.432
7.3750	.929	.455
7.6250	.929	.500
8.2500	.953	.568
9.2500	.988	.659
10.2500	1.000	.909
11.7500	1.000	1.000
